# Visceral Adiposity Index in Breast Cancer Survivors: A Case-Control Study

**DOI:** 10.1155/2020/8874916

**Published:** 2020-12-09

**Authors:** Elías Cardoso-Peña, Alexandra E. Soto Pina, Ángel Gómez Villanueva, Gerardo Emilio López Chavez, Pablo Ramírez Martínez, Humberto Ramírez Montoya, María Guadalupe Berumen Lechuga, Alejandra Donají Benitez Arciniega, María de Lourdes Alarcón Fortepiani, Roxana Valdés Ramos, José de Jesús Garduño García

**Affiliations:** ^1^Family Medicine Unit No. 220, Mexican Institute of Social Security, Toluca, Mexico; ^2^School of Medicine, Autonomous University of the State of Mexico, Toluca, Mexico; ^3^Regional General Hospital No. 251, Mexican Institute of Social Security, Metepec, Mexico; ^4^Delegation of the State of Mexico West, Mexican Institute of Social Security, Toluca, Mexico; ^5^Rosenberg School of Optometry, University of the Incarnate Word, San Antonio, TX, USA; ^6^Department of Cellular and Integrative Physiology, UT Health, San Antonio, TX, USA

## Abstract

**Background:**

Breast cancer (BC) is the first cause of cancer morbidity and mortality in women. This disease has been linked to obesity; however, it is not clear how fat accumulation affects women who survive breast cancer. Although the visceral adiposity index (VAI) is a marker of cardiometabolic risk and adipose tissue dysfunction, it is not clear how it changes in breast cancer survivors. The aim of this investigation was to compare VAI in women with and without breast cancer.

**Methods:**

A case-control cross-sectional study was conducted on women who were BC survivors and women without the history of BC (control group). Body composition was assessed using electrical bioimpedance while VAI by means of waist circumference (WC), body mass index (BMI), triacylglycerols (TG), and high-density lipoprotein cholesterol (HDL-C).

**Results:**

49 women in the BC survivor group and 50 in the control group. WC was wider in the survivor group as regards control (93.65 ± 10.48 vs. 88.52 ± 9.61 cm) (*p*=0.025); at once, TG and VAI were significantly higher for the survivor group (243.55 ± 199.84 vs. 159.84 ± 75.77) (*p*=0.007) and (11.03 ± 11.15 vs. 6.41 ± 3.66) (*p* < 0.005), respectively. Body composition parameters were similar in both groups.

**Conclusions:**

VAI is higher in women who are BC survivors in comparison with controls matched by age and bodyweight.

## 1. Introduction

Breast cancer (BC) is the second most frequent neoplasm among the general population worldwide, only surpassed by lung cancer; however, it is the first cause of cancer morbidity and mortality in women [1, 2]. Smoking, fat-rich diet, high estrogen levels, obesity, alcohol consumption, hormone therapy, advanced maternal age, and late menopause are some of the known breast cancer risk factors [3–6]. Some of these risk factors are related to insulin resistance [7] and obesity [8].

Obesity is the excessive accumulation of fat in adipose tissue, with a positive energy imbalance and weight gain. Types of fat include visceral central and peripheral subcutaneous [9, 10]. The risk of developing cancer increases 1.6 times with a high body mass index (BMI) and the presence of visceral adiposity [11]. However, the relationship between BC and obesity is complex, as both are influenced by age, menopause, and the expression of estrogen and progesterone receptors [9, 12]. Moreover, the conditions above are characterized by a chronic inflammatory state, and obesity can promote further inflammation in cancer by increasing the production of proinflammatory cytokines [13, 14].

In the majority of studies, obesity is ascertained resorting to BMI. This parameter cannot differentiate between visceral and peripheral fat. Interestingly, visceral adiposity index (VAI) is a marker of adipose tissue dysfunction, which comprises physical and biochemical parameters such as WC, BMI, triacylglycerols (TG), and high-density lipoprotein cholesterol (HDL-C) [15–17]. VAI has been associated with metabolic syndrome and cardiovascular and cerebrovascular diseases, particularly in men and advanced age; such an association is not reported for WC and BMI [18].

Breast cancer survivors with high fat mass have a higher risk of relapse and developing cancer in other parts of the body as well. Furthermore, these patients experience adverse effects induced by chemotherapy or radiation, including increased fat mass, adding to nausea, vomiting, energy loss, fatigue, and muscle mass loss [19, 20]. It is not clear, nevertheless, how visceral adiposity changes upon the development of BC and its therapeutic process. Therefore, our hypothesis was that VAI, a specific marker of central adiposity, as well as metabolic syndrome components are higher in BC survivors than in matched controls.

## 2. Materials and Methods

A case-control cross-sectional study was performed. Participants were recruited at the General Regional Hospital 251 of the Mexican Institute of Social Security (IMSS). The investigations included women between 40 and 65 years of age who were categorized in two groups. The first included women with breast cancer diagnostic at the follow-up, with at least one year without any BC-related chemotherapy with cytotoxic drugs, radiotherapy, or surgery, who attended regular oncology appointments; a review of their medical files was carried out in order to obtain general data and define all the inclusion and exclusion criteria. Patients with known type 2 diabetes, hypertension, kidney or hepatic failure, or any other chronic disease, which the research group considered could modify the variables under study, were excluded from participating in the study. The second group (control) comprised women who attended the hospital for annual mammography screening and were matched by age with the survivor group. The absence of BC was determined by including women with BIRADS 1, 2, and 3 only. Patients with diabetes mellitus, hypertension, and kidney or hepatic failure were excluded from either group. Women who met these criteria were contacted and invited to participate, and individual appointments were scheduled.

### 2.1. Evaluation of Body Composition and Biochemical Analysis

Body composition was assessed by electrical bioimpedance using an InBody® 230 apparatus (InBody Co., Seoul, South Korea). This analysis included 10 measurements at 2 different frequencies, 20 kHz and 100 kHz, in 5 segments (right arm, left arm, trunk, right leg, and left leg). Participants were fasting, barefoot, and wearing light clothing when taking these measurements. The data obtained were total body water (TBW), proteins, minerals, body fat mass (BFM), bodyweight (BW), skeletal muscle mass (SMM), BMI, percentage of body fat (PBF), lean segmental mass (LSM), trunk segmental fat (TSF), obesity analysis (BMI, PBF), fat free mass (FFM), WHiR, visceral fat level (VFL), and segment impedance and frequency. Height was measured using a stadiometer (Seca® 213) with a 20–205 cm range.

### 2.2. Laboratory Procedures

Blood samples were taken with BD Vacutainer® Tubing SSTTM and EDTA K2 tubes. Once the sample was obtained, measurements were performed on an AU680 Beckman Coulter® device. HDL-C was determined with selective detergent methods; TG was determined with the glycerol phosphate oxidase test; low-density lipoprotein cholesterol (LDL-C) concentrations were calculated with the Martin–Hopkins equation, while glucose was evaluated with the hexokinase-UV/NAD test. HbA1c was measured by HPLC (high-performance liquid chromatography) utilizing a Variant II Turbo® system. All the tests were run by Quest Diagnostics® Clinical Analysis Laboratories (Mexico).

VAI was calculated with the following equation: [WC/(36.58+(1.89 × BMI))] × [TG/0.81] × [1.52/HDL − C] [15].

### 2.3. Statistical Analysis

A descriptive analysis was carried out for continuous variables through means and standard deviation. The normal distribution of the variables was found using the Kolmogorov–Smirnov test. The comparison of the means between the groups was by means of the *t*-test for variables that followed a normal data distribution, while Mann–Whitney *U* if they did not. A chi-squared test (*χ*^2^) was also run to find associations between qualitative variables. The statistical analysis was carried out with IBM® SPSS® Statistics, Mac version 25.

## 3. Results

A total of 56 women were enrolled in the BC survivor group and 54 women in the matched control group. However, 11 women (7 in the survivor group and 4 in the control) were excluded owing to the presence of diabetes mellitus diagnosed upon the study appointment. The final sample included 49 women in the BC survivor group and 50 controls. The most frequent histological subtype was ductal (83.7%), whereas the molecular subtype was luminal A (49.0%) (Table 1). 16 out of the 49 breast cancer patients were taking tamoxifen (32.7%), letrozole (18.4%), anastrozole (6.1%), and 21 no medication (42.9%). Control patients were free of any chronic medication.

**Figure 1:**
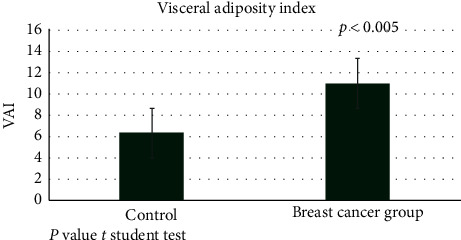
Visceral adiposity index in breast cancer survivors.

Age, marital status, and personal BC history were not significantly different between groups (Table 2). Interestingly, WC was wider in the survivor group than in the control one (*p*=0.025), though there were no differences in BMI and BW. With regards to body composition, there were no differences in bioimpedance parameters nor in blood concentrations of glucose, HbA1c, HDL-C, LDL-C, and total cholesterol. However, TG concentrations were significantly higher in the survivor group as compared with controls (Table 3); in addition, VAI was also significantly higher in the group of survivors compared with controls (Figure 1).

## 4. Discussion

In this study, we found that BC survivors have a higher VAI than the control group, which is consistent with our hypothesis. These data suggest that BC survivors may have different early changes in their metabolic profile enhancing a predisposed condition for a future risk of diabetes or a cardiovascular disease [20].

Body composition has been associated with metabolic dysfunction, while increased body mass with changes in the physiological function of adipose tissue, inflammatory mediators, and chronic inflammation as well. Such factors have also been related to various cancers. Insulin resistance seems to be a possible link between obesity and cancer [21]. Even breast cancer has many well-described risk factors owing to which insulin resistance could play an important role in the disease [22].

Measuring insulin resistance in clinical daily practice is still a big challenge. Hyperglycemic hyperinsulinemic clamp is recognized as the gold standard method to measure insulin resistance, albeit it is a complex method that is only used in the sphere of research [23]. There exist various methods to assess insulin resistance with insulin measurements such as HOMA IR; yet, as in the case of insulin measurement, it is not readily available in most places. For this reason, surrogate indexes of insulin sensitivity have been proposed by means of affordable anthropometric and/or laboratory measures routinely gathered in clinical settings [24]. In the present study, we use VAI in the assessment of indirect insulin resistance measurement, as it has shown a good correlation with the gold standard method [25]. In clinical settings, it might be very useful, as it enables a better overview of metabolic health. Obesity and other related markers have been associated with the risk of BC recurrence [26–28]. However, this is the first study to show that VAI could be an important factor to assess in BC survivors.

Metabolic syndrome, with its various components, is a risk factor for BC recurrence; hence, it may be considered a recurrence predictor and be included as a part of diagnostic standards [29]. In this research, 59.2% of the survivors and 41.3% of the controls presented metabolic syndrome, which agrees with another study on Mexican women, in which the frequency was 57% in BC survivors and 36% in women without personal BC history [30].

Furthermore, other variables related to central obesity such as WC also increased in the BC survivor group, a result that concurs with VAI data. The advantage of using VAI is that it is a more complete index than WC, since it includes biochemical and physical variables to be computed, which produces a more accurate diagnosis of the nutritional status and metabolic syndrome presence or risk in this type of patients. This could be helpful to improve interventions or lifestyle modifications in BC survivors and prevent chronic diseases from developing in healthy women.

In the present study, the percentages of SMM, FFM, TBW, proteins, and minerals decreased, while BFM and SFT increased in the survivors when compared to controls, and they were not statistically significant though. Regardless of their statistical significance in this study, changes in these parameters have shown to contribute to decrease the patients' quality of life [31, 32] and increase the risk of cancer [33]. We also found that BFM for BC survivors is 41.12%, concurring with data reported by Lee et al. (40.4%), using the same electrical bioimpedance technique [34].

These results bear similarities with those obtained in prospective studies performed with dual-energy X-ray absorptiometry (DEXA) for 12–15 months, which do not report differences between the body composition of surviving women and that of the general population nor between the types of therapy they received after the diagnosis of cancer [20, 26, 35]. By contrast, another report shows that electrical bioimpedance underestimates body fat percentage in comparison with DEXA [36]. This may be a limitation of this approach as we only used 2 frequencies to perform the electrical bioimpedance analysis.

The concentrations of blood lipids which have been associated with metabolic syndrome, cardiovascular disease, and atherosclerosis [37, 38] are elevated in survivor women. Our findings show that the survivors' lipid profile differs from that of controls. While HDL-C and LDL-C are not significantly different between the groups, TG are significantly elevated in the survivor group. However, the concentrations of HDL-C have an impact on the atherogenic index and VAI; this way, in survivor women, such levels are implicitly important.

Another study conducted on women, with either a recent diagnosis of BC or without it (healthy women), reported no significant differences in lipid concentrations [39]. Even though there are reports of HDL-C and TG association with BC, specifically by promoting cell proliferation, migration, and angiogenesis [40, 41], this association cannot be disregarded.

In Mexico, where our study was performed, there is a strong correlation between VAI and TG. Noticeably, elevated TG levels have been reported for Mexican population (adolescents and adults) [42, 43]. It may be even associated with polymorphisms of this lipoprotein gene; however, it needs to be corroborated by means of more specific studies. Although there is no difference between the two groups, it may be necessary to research a much larger group of subjects, as it could serve as a marker for cancer diagnosis and relapses.

In this group of survivors, the most frequent BC subtypes were histologically ductal carcinoma and molecular luminal A type. Other studies have reported an association between obesity and BC with positive estrogen receptors, which was unrelated to other molecular subtypes [44].

No association was found between VAI and obesity with BC with positive estrogen receptors in the group of survivors. To further dissect this possible association, additional studies should be performed on populations in which metabolic syndrome and obesity are less prevalent or the individual components as well as the level of metabolic syndrome and obesity are assessed.

This research presents some limitations, for instance, a larger sample size of breast cancer survivors would be needed to identify strong associations between metabolic syndrome and breast cancer and establish thus a clearer relationship between VAI and its components with BC. Another limitation is the cross-sectional design of the study, which does not allow for a follow-up of BC survivors and establish whether there is a risk of either BC relapse or metabolic syndrome when VAI is elevated.

However, adhering to a healthy diet with high consumption of vegetables and fruits and reducing overweight and obesity will bring about additional beneficial effects in BC survivors by reducing the risk of relapse, comorbidities, and death [18]. In this sense, the inclusion of VAI in their follow-up becomes imperative.

The conclusion of this study is that VAI increases in BC survivors compared with controls, regardless of BMI. Early identifications of this marker may be useful as a tool in future studies to assess increments in the risk of metabolic syndrome and cardiovascular diseases among this population.

## Figures and Tables

**Table 1:** Histological and molecular BC subtypes.

	Frequency	Percentage
Histopathological BC subtype		
Ductal	41	83.7
Lobular	5	10.2
Mucinous	1	2
Mixed	1	2
Undifferentiated	1	2

Molecular BC subtype		
Not performed	6	12.2
Luminal A	24	49
Luminal B	11	22.4
Her-2	5	10.2
Triple negative	3	6.1

**Table 2:** General characteristics of controls and BC survivors.

	Total	Controls	Survivors	*p* value
Age	50.77 ± 6.84	49.96 ± 6.66	51.59 ± 6.99	0.236^*∗*^
Marital status (married)	69 (69.7%)	38 (76%)	31 (63.3%)	0.168
Family history of breast cancer	8 (8.1%)	3 (6%)	5 (10.2%)	0.443
Family history of type 2 diabetes	58 (58.6%)	26 (52%)	32 (65.3%)	0.179
Family history of hypertension	22 (22.2%)	11 (22%)	11 (22.4%)	0.957

Mean and standard deviation, frequencies, *t*-test, and *χ*^2^; ^*∗*^Mann–Whitney *U*, *p* < 0.05.

**Table 3:** Anthropometric characteristics, body composition, and biochemical markers in controls and BC survivors.

	Controls	Survivors	*p* value
BW (kg)	67.20 ± 8.93	66.13 ± 10.21	0.579
BMI (kg/m^2^)	27.560 ± 3.84	28.32 ± 3.98	0.331
WC (cm)	88.52 ± 9.61	93.65 ± 10.48	0.025^*∗*^
BFM (%)	39.55 ± 7.43	41.12 ± 5.13	0.225
SMM (%)	32.89 ± 4.14	31.94 ± 2.81	0.182
TSF (%)	40.16 ± 7.24	41.32 ± 4.89	0.351
FFM (%)	60.34 ± 7.59	58.90 ± 5.15	0.271
TBW (%)	44.33 ± 5.44	43.27 ± 3.75	0.265
Proteins (%)	11.89 ± 1.49	11.61 ± 1.03	0.277
Minerals (%)	4.20 ± .53	4.01 ± .41	0.076^*∗*^
Glucose (mg/dL)	92.87 ± 15.63	94.96 ± 16.66	0.394^*∗*^
HbA1c (%)	5.67 ± .43	5.78 ± .56	0.207^*∗*^
TG (mg/dL)	159.84 ± 75.77	243.55 ± 199.84	0.007^*∗*^
HDL-C (mg/dL)	49.66 ± 10.27	47.29 ± 10.27	0.323^*∗*^
LDL-C (mg/dL)	118.61 ± 36.25	121.96 ± 35.06	0.571^*∗*^

BW, body weight; BMI, body mass index; WC, waist circumference; BFM, body fat mass; SMM, skeletal muscle mass; TSF, trunk segmental fat; FFM, fat free mass; TBW, total body water; HbA1c, glycosylated hemoglobin; TG, triacylglycerols; HDL-C, high-density lipoprotein cholesterol; LDL-C, low-density lipoprotein cholesterol; VLDL-C, very low-density lipoprotein cholesterol. *t*-test; ^*∗*^Mann–Whitney *U* test, *p* < 0.05.

## Data Availability

The data used to support the findings of this study are available from the corresponding authors upon request.
